# Supplementary material for: CTHRC1 Expression Results in Secretion-Mediated, SOX9-Dependent Suppression of Adipogenesis: Implications for the Regulatory Role of Newly Identified CTHRC1+/PDGFR-Alpha+ Stromal Cells of Adipose
Source: Int J Mol Sci. 2025 Feb 20;26(5):1804. doi: 10.3390/ijms26051804 (PMC11898434; doi:10.3390/ijms26051804)
Supplement: Supplementary file 1 [file ijms-26-01804-s001.zip › ijms-3457092-supplementary.pdf]

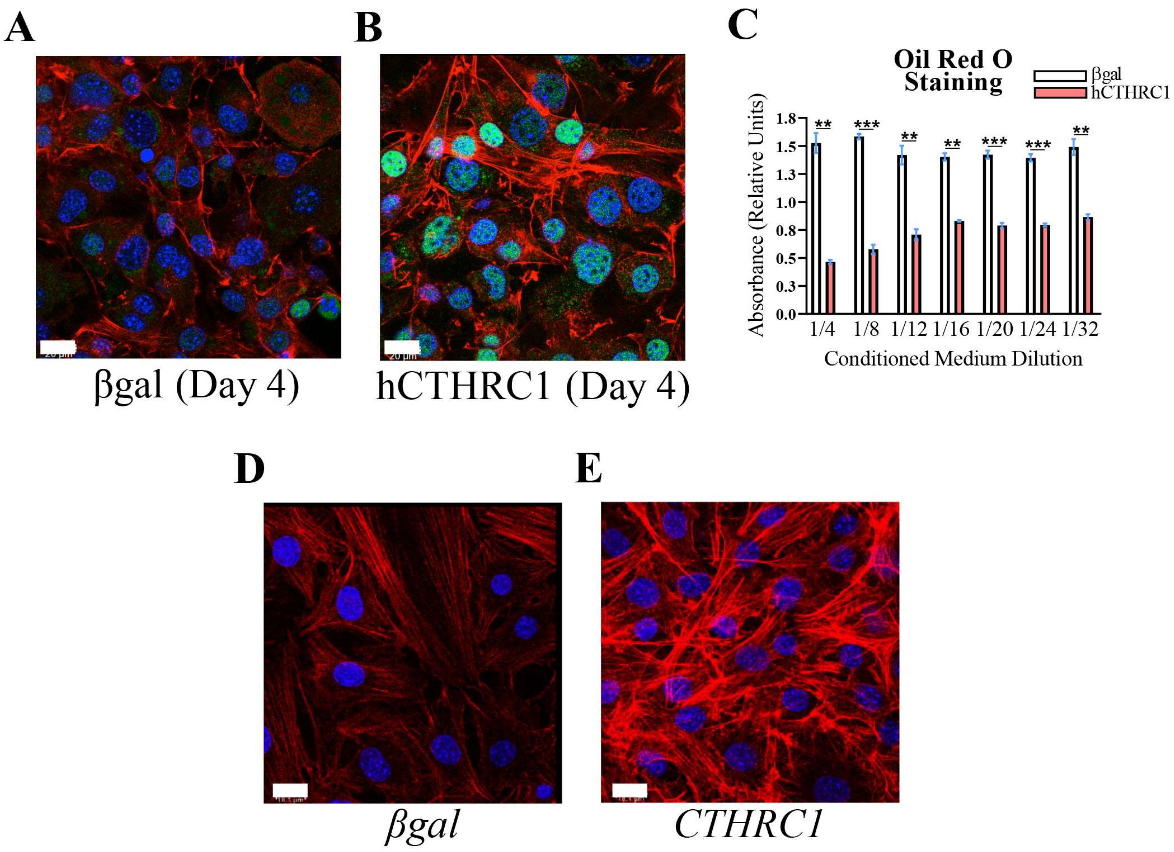

Supplemental Figure S1. **Supplementary Supporting Data**. **A**,**B**) Representative confocal microscopy images of SOX9 protein localization on Day 4 of adipogenic differentiation in 3T3-L1 cells treated with either βgal conditioned medium (**A**) or hCTHRC1 conditioned medium (**B**) at a 1/4 dilution: nuclei (blue); SOX9 (green); F-actin/Alexa Fluor 546 Phalloidin (red). Scale bar: 20 µm. **C**) Representative Oil Red O quantification data. 3T3-L1 cells were seeded on Day -3 with βgal or hCTHRC1 conditioned medium at the following dilutions: 1/4, 1/8, 1/12, 1/16, 1/20, 1/24, or 1/32. Cells were seeded in a 24-well plate corresponding to three wells per each conditioned medium dilution, and then chemically stimulated to undergo adipogenic differentiation beginning on Day 0. Cells were formalin fixed on Day 6 and stained with Oil Red O, which was then eluted and its concentration determined by absorbance spectroscopy (** $p \leq 0.01$, *** $p \leq 0.001$). **D**,**E**) Representative confocal microscopy images of the enhanced F-actin cytoskeleton commensurate to the overexpression of human CTHRC1. 3T3-L1 cells were transduced with adenoviral vectors overexpressing either control *β–galactosidase* (**D**) or human *CTHRC1* (**E**). Two days following the onset of adenoviral transduction, cells were formalin fixed and treated with Hoechst nuclear stain (blue) and Alexa Fluor 546 Phalloidin (red). Scale bar: 20 µm.

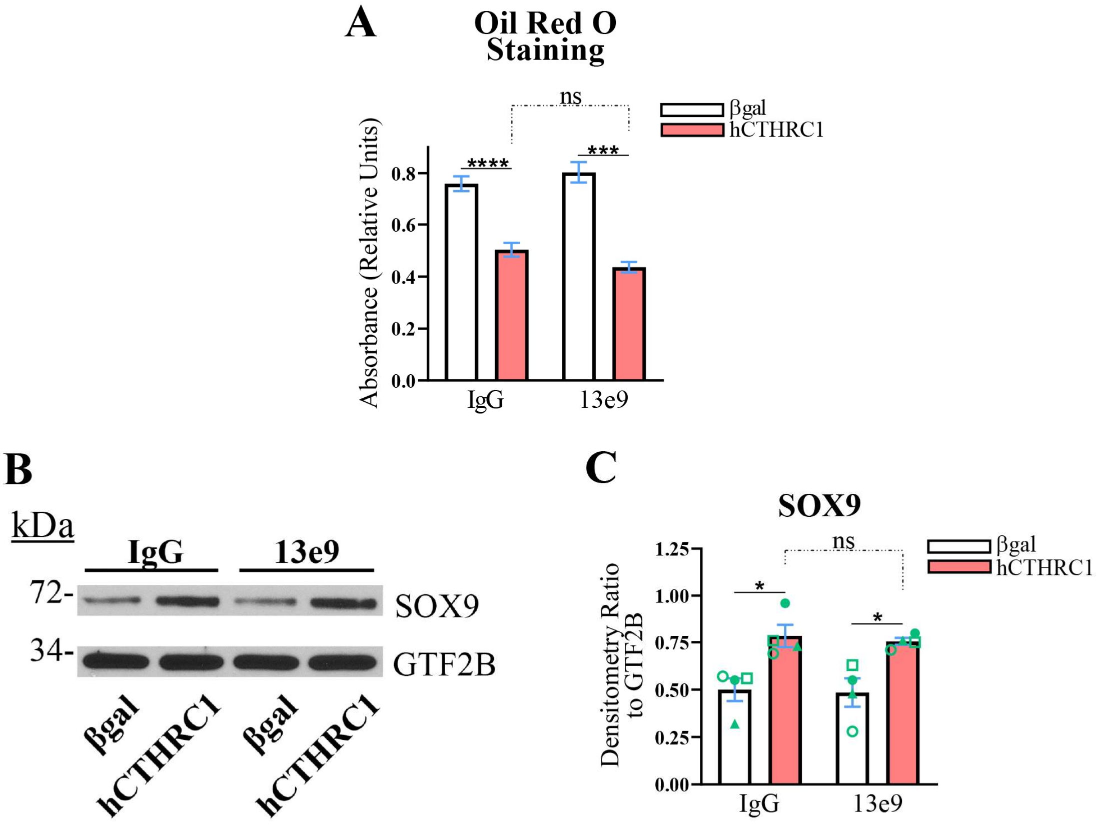

Supplemental Figure S2. **The Anti-Adipogenic Effect of hCTHRC1 Conditioned Medium Is Unaffected by Depletion of Human CTHRC1 Protein Levels**. Protein A Sepharose was conjugated to either mouse anti-CTHRC1 IgG (13e9) or naïve mouse IgG (IgG). hCTHRC1 and control βgal conditioned medium were then incubated with 13e9- or IgG-Protein A Sepharose conjugates, following which the supernatants were collected and used for study. 13e9 conjugated to Protein A Sepharose depleted human CTHRC1 protein expression levels in hCTHRC1 conditioned medium by greater than 80% based on quantification using an established ELISA. 3T3-L1 cells were treated with respective conditioned media at a 1/60 dilution beginning on Day -3 and subsequently throughout the six-day period of chemically stimulated adipogenic differentiation. Respective conditioned media were freshly applied each day of the experiment. After six days of differentiation, cells were formalin fixed and stained with Oil Red O (**A**), which was then eluted and its concentration determined by absorbance spectroscopy (n=4; *** $p \leq 0.001$, **** $p \leq 0.0001$). Not statistically significant (ns). Whole-cell lysates were collected on Day 0 to determine SOX9 protein expression levels by Western blot analysis (**B**). **C**) Average SOX9 protein fold change densitometry values relative to housekeeping GTF2B protein expression levels from four independent experiments (n=4; * $p \leq 0.05$). Green symbols (triangle, square, open circle, and closed circle) are paired according to experimental replication. Not statistically significant (ns).

Table of Two-Way ANOVA				
Experiment Identifier	Sum of Squares	Mean Square	F value	Significance (p-value)
A	0.0202	0.0202	6.492	0.0215
B	0.0436	0.0436	5.779	0.0272
C	0.0844	0.0844	27.231	<0.0001
D	0.0342	0.0342	7.757	0.0114

Supplemental Table S1. **Table of Two-Way ANOVA to Figure 5F**. Two-way analysis of variance (ANOVA) table displaying the significance of the interaction term between the "vehicle" and "N+Y" groups graphically displayed in Figure 5F. The significant interaction term in four independent experiments strongly supports the hypothesis that the anti-adipogenic effect of hCTHRC1 conditioned medium is diminished in a statistically significant manner when specific Rho-like GTPase chemical inhibitors are applied (*i.e.*, N and Y). N (NSC 23766) is a well-defined Rac1 activation-specific inhibitor [19], and Y (Y-27632) is a potent inhibitor of the direct Rho effector, Rho-associated kinase [20].
